# Dorsomedial Ventromedial Hypothalamic Nucleus Growth Hormone-Releasing Hormone Neuron Steroidogenic Factor-1 Gene Targets in Female Rat

**DOI:** 10.1080/17590914.2024.2403345

**Published:** 2024-10-14

**Authors:** Subash Sapkota, Sagor C. Roy, Karen P. Briski

**Affiliations:** School of Basic Pharmaceutical and Toxicological Sciences, College of Pharmacy, University of Louisiana Monroe, Monroe, LA, USA

**Keywords:** GAD_65_, Ghrh, insulin-induced hypoglycemia, neuronal nitric oxide synthase, sex differences, SF-1

## Abstract

The prospect that the ventromedial hypothalamic nucleus (VMN) transcription factor steroidogenic factor-1/NR5A1 (SF-1) may exert sex-dimorphic control of glucose counterregulation is unresolved. Recent studies in male rats show that SF-1 regulates transcription of co-expressed hypoglycemia-sensitive neurochemicals in dorsomedial VMN growth hormone-releasing hormone (Ghrh) neurons. Gene knockdown and laser-catapult-microdissection/single-cell multiplex qPCR techniques were used here in a female rat model to determine if SF-1 control of Ghrh neuron transmitter marker, energy sensor, and estrogen receptor (ER) variant mRNAs varies according to sex. Data show that in females, hypoglycemia elicits a gain of SF-1 inhibitory control of VMNdm Ghrh neuron Ghrh and Ghrh-receptor gene profiles and loss of augmentation of glutaminase transcription; SF-1 gene silencing diminished eu- and hypoglycemic patterns of neuronal nitric oxide gene transcription. SF-1 imposes divergent control of baseline and hypoglycemic glutamate decarboxylase_65_ (GAD)-1 (stimulatory) versus GAD2 (inhibitory) mRNAs in that sex. SF-1 stimulates baseline VMNdm Ghrh neuron PRKAA1/AMPKα1 and PRKAA2/AMPKα2 gene expression, yet causes opposite changes in these gene profiles during hypoglycemia. SF-1 exerts glucose-dependent control of ER-alpha and G-protein-coupled ER-1 transcription, but blunts ER-beta gene profiles during eu- and hypoglycemia. In females, SF-1 knockdown did not affect hypercorticosteronemia or hyperglucagonemia, but blunted hypoglycemic suppression of growth hormone secretion. Results show that SF-1 expression is critical for female rat VMNdm Ghrh neuron counterregulatory neurochemical, AMPK catalytic subunit, and ER gene transcription responses to hypoglycemia. Sex differences in direction of SF-1 control of distinctive gene profiles may result in observed disparities in SF-1 regulation of counterregulatory hormone secretion between sexes.

## Introduction

Steroidogenic factor 1 (SF-1)/NR5a1 is a nuclear receptor superfamily member that directs the development and function of the hypothalamus-pituitary-steroidogenic organ axis, namely the ventromedial hypothalamic nucleus (VMN), adrenal glands, and gonads (Kim et al., 2009; McClellan et al.,^2006^; Schimmer & White, 2010). The VMN is a vital component of a distinctive sex-dimorphic network in the brain that is established by estrogen receptor (ER) imprinting during the ‘critical period’ of central nervous system development (Simerly, 2002). The VMN exerts sex-specific control of numerous functions, including sexual and maternal behaviors, physical activity, and metabolic homeostasis (Correa et al., 2015; Hashikawa et al., 2017; Martínez de Morentin et al., 2014; Musatov et al., 2007; Yang et al., 2013). Cellular energy balance is continuously monitored in the VMN and other select neural loci by energy-screening interoceptive neurons that adjust synaptic firing in accordance with changes in metabolic fuel provision (Adachi et al., 1995, 1997; Ashford et al., 1990; Oomura et al., 1969; Silver & Erecińska, 1998). This dynamic readout, along with hormonal surrogate signals for peripheral glucose metabolism, energy storage volume, and satiety (Flak et al., 2020; Meek et al., 2013; Sabatier & Leng, 2010), is utilized by the VMN to regulate autonomic, neuroendocrine, and behavioral outflow that preserves glucose homeostasis. Characterized glucose counterregulation-enhancing or -repressive neurotransmitter marker proteins expressed in the VMN exhibit sex-dimorphic responses to insulin-induced hypoglycemia (IIH), suggesting that VMN input to the extensive neural glucostatic circuitry (Watts & Donovan, 2010) may be dissimilar in the two sexes (Alshamrani et al., 2020).

Central SF-1 actions regulate systemic energy and glucose homeostasis (Choi et al., 2013; Garfield et al., 2014; Kim et al., 2011; Meek et al., 2016; Xu et al., 2011; Zhang et al., 2008). In the brain, SF-1 is expressed exclusively in neurons located in the dorsomedial VMN (VMNdm) (Cheung et al., 2013; Kim et al., 2019). Apart from substantiation that SF-1 co-exists with VGLUT2 (Tong et al., 2007), a marker for glutamate (Glu) neurotransmission (Moriyama & Yamamoto, 2004), insight on the neurochemical phenotype of neurons that implement SF-1 regulatory actions is limited. Contemporary studies, using combinative *in situ* immunocytochemistry/laser-catapult-microdissection/single-cell multiplex qPCR methodology together with a validated *in vivo* experimental model for IIH (Paranjape & Briski, 2005), showed that VMNdm growth hormone-releasing hormone (Ghrh) neurons contain hypoglycemia-sensitive SF-1 mRNA, and that Ghrh neurotransmission is crucial for hypoglycemic counterregulatory hormone secretion patterns (Sapkota et al., 2023). These neurons also contain gene transcripts that encode enzyme markers for non-neuropeptide glucose-regulatory neurotransmitters [the labile gas nitric oxide (NO) and the amino acids γ-aminobutyric acid (GABA) and Glu], and are subject to neuro-modulatory control by Ghrh receptor (Ghrh-R) signaling. Hypothalamic AMPK supplies critical cues on cellular energy stability to neural pathways that control systemic energy balance (López, 2018; Pimentel et al., 2013; Xue & Kahn, 2006). Ventromedial hypothalamic AMPK is implicated in neural regulation of hypoglycemic patterns of counterregulatory hormone secretion (Han et al., 2005; McCrimmon et al., 2008). We reported that SF-1—immunoreactive VMN neurons contain the catalytic alpha subunit of the ultra-sensitive energy gauge 5’-AMP-activated protein kinase (AMPK), and that this subunit protein is phosphorylated in response to IIH (Ibrahim et al., 2020). Recent observations that VMNdm Ghrh neurons express PRKAA1 and PRKAA2, genes that yield the AMPK catalytic subunit isoforms alpha1 and alpha2, affirm the prospect that this neuron population engages in metabolic sensory function (Sapkota & Briski, 2024). Developmental organization of the VMN results in sex-specific estradiol control of VMN functions. Evidence that VMNdm Ghrh neurons exhibit gene transcripts for ER variants, i.e. the nuclear ERs ER-alpha (ERα) and ER-beta (ERβ) and the plasma membrane G protein-coupled ER-1 (GPER), supports the likelihood that this neuron population may be a substrate for integration of SF-1 and estradiol signaling.

Co-transmission of neurochemicals of diverse chemical structure, spatial, and temporal profiles that impose distinctive control of counterregulatory hormone release may underlie a capacity of VMNdm Ghrh neurons to contribute complex, coordinated multi-modal input to the brain glucostatic circuitry. A key ensuing question is whether such input is controlled by SF-1. Recent research used *in vivo* gene knockdown tools for targeted repression of VMN SF-1 gene expression in the male rat (Sapkota et al., 2024) to acquire proof of concept that SF-1 may regulate transcription of genes that encode Ghrh and/or protein markers for co-expressed glucose-regulatory neurotransmitters. Emerging documentation of discriminative SF-1 control of VMNdm Ghrh neuron counterregulatory neurochemical and ER variant gene transcription together with governance of basal and hypoglycemic counterregulatory endocrine profiles infers that these cells are effectors of SF-1 direction of glucose counterregulation in the male rat. Current studies addressed the possibility that SF-1 may regulate Ghrh neuron receptivity to estradiol and release of distinctive neurochemicals during glucose homeostasis and/or systemic deficiency according to sex. Here, single VMNdm Ghrh neurons were collected by laser-catapult-microdissection from control versus SF-1 siRNA-pretreated, vehicle- or insulin- (INS)-injected female rats for multiplex qPCR analysis of panels of genes encoding glucose-regulatory neurochemical marker, AMPK alpha subunit isoforms, and nuclear and membrane ER variants. The discussion below pertaining to the significance of outcomes of current research involving female rats includes commentary on similarities and differences compared to similar work focused on SF-1 regulation of similar experimental endpoints in the male (Sapkota et al., 2024).

## Materials and Methods

### Animals

Adult female Sprague Dawley rats (2–3 months of age; mean 250 g *bw*) were housed in groups of 2–3 per shoe-box cage, under a 14-hr light: 10-hr dark cycle; lights on at 05.00 hr. Animals were acclimated to handling prior to experimentation. Animals had unrestricted access to standard laboratory chow and tap water. Study protocols and procedures were carried out in compliance with the NIH Guide for Care and Use of Laboratory Animals, 8th Edition, under ULM Institutional Animal Care and Use Committee approval.

### Experimental Design

On Study Day 1, animals were randomly assigned to 4 treatment groups (*n* = 6 per treatment group). Rats were anesthetized by intraperitoneal injection of ketamine/xylazine (9.0 mg ketamine/1.0 mg xylazine/0.1 mL/100g *bw*) in preparation for bilateral intra-VMN injection (total volume: 1.0 μL; infusion rate: 3.6 μL/min; coordinates: −2.5 mm posterior to bregma, 0.6 mm lateral to midline, 9.0 mm ventral to skull surface) of scramble (SCR) siRNA (500 pmol; Accell Control Pool Non-Targeting; prod. no. D-001910-10-20; Horizon Discovery, Waterbeach, UK) or SF-1 siRNA (500 pmol; Accell siRNA rat SF-1, set of 4; prod. no. EQ-100091-00-0010; Horizon Disc), as reported (Sapkota et al., 2024). Injections were made using a 33 gauge Neuros syringe (prod. no. 53496; Stoelting Co., Wood Dale, IL), under Neurostar stereotactic Drill and Injection Robot (Neurostar, Tubingen, Germany) control. At the conclusion of surgery, rats were injected with ketophen (*sc*; Zoetis Inc., Kalamazoo, MI) and enrofloxacin (IM; Bayer HealthCare LLC, Animal Health Division, Shawnee Mission, KS), and treated by topical 0.25% bupivacaine application to closed incisions. After full recovery from anesthesia, animals were transferred to single-occupancy cages. On Study Day 7, animals were injected *sc* at 09.00 hr with vehicle (V; sterile diluent; Eli Lilly & Co., Indianapolis, IN) or neutral protamine Hagedorn insulin (INS; 10.0 U/kg *bw* (Napit et al., 2019); Eli Lilly); animals were sacrificed by rapid decapitation one hr post-injection. Each brain was dissected whole, then snap-frozen by immersion in liquid nitrogen-cooled isopentane for storage at -80 °C. Brains were cut into 10 μm- or 100 μm-thick frozen sections, over alternating distances of 100 μm (1 × 100 μm sections) and 100 μm (10 × 10 μm thin sections), respectively, between −1.80 and −2.3 mm posterior to *bregma*, for single-cell multiplex quantitative reverse transcription PCR (RT-qPCR) analysis of VMNdm Ghrh neurons or Western blot analysis of micropunch-dissected VMNdm tissue, respectively.

### Laser-Catapult-Microdissection of VMNdm Ghrh Neurons

Individual 10 μm-thick fresh-frozen sections were mounted on polyethylene naphthalate membrane-coated slides (prod. no. 415190-9041-000; Carl Zeiss Microscopy LLC, White Plains, NY). Tissues were fixed with ice-cold acetone (5 min) prior to blocking (2 hr) with 1.5% normal goat serum (prod. no. S-2000, Vector Laboratories, Burlingame, CA) in Tris-buffered saline, pH 7.4, (TBS), 0.05% Triton X-100. (2 hr). After incubation with a rabbit anti-preproGhrh primary antiserum (prod. no. PA5-102738, 1:2000; Invitrogen, Waltham, MA) (48–72 hr; 4 °C), as described (Sapkota et al., 2023), sections were then exposed to a horseradish peroxidase-labeled goat anti-rabbit secondary antibody (prod. no. PI-1000, 1:1000; Vector Lab.; 1 hr), followed by ImmPACT 3,3-diaminobenzidine peroxidase substrate kit reagents (prod. no. SK-4105; Vector Lab.). For each animal, individual neurons exhibiting cytoplasmic Ghrh-immunoreactivity (ir) were detached and propelled from tissue sections using a Carl Zeiss P.A.L.M. UV-A microlaser IV system, as reported (Sapkota et al., 2023). Each cell was collected in an adhesive cap (prod. no. 415190-9181-000; Carl Zeiss) containing lysis buffer (4 µL; Single Shot Cell Lysis Kit, prod. no. 1725080; Bio-Rad Laboratories, Hercules, CA) for multiplex gene expression analysis.

### Single-Cell Multiplex Quantitative RT-qPCR Analysis

#### Complementary DNA (cDNA) Synthesis and Amplification

Single-cell lysates were centrifuged (3,000 rpm; 4 °C) prior to sequential incubation at 25 °C (10 min) or 75 °C (5 min) in a Bio-Rad iCyclerQ RT-PCR Detection System. Sample RNA integrity, purity, and quantity were analyzed using a ThermoFisherScientific (Waltham, MA) NanoDrop One^c^ microvolume UV-Vis spectrophotometer. Single-cell mRNA samples were reverse-transcribed to cDNA by addition of cDNA synthesis buffer (1.5 µL; Script^TM^ Advanced cDNA Synthesis Kit. prod. no. 1725038; Bio-Rad) prior to initial 46 °C (20 min) incubation, followed by secondary 95 °C (1 min) incubation, using described methods (Ali et al., 2022a, 2022b; Alshamrani et al., 2022). Pre-amplification master mix preparation involved combining PrimePCR^™^ PreAmp for SYBR Green Assays for SF-1/NR5A1 (prod. no. qRnoCID0001458), Ghrh (prod. no. qRnoCID0007723), glutamate decarboxylase (GAD)_67_/GAD1 (prod. no. qRnoCID0004554), GAD_65_/GAD2 (pro. no. qRnoCID0003485), NOS1/nNOS (prod. no. qRnoCED0009301), glutaminase (GLS) (prod. no. qRnoCID0007756), PRKAA1/AMPKα1 (prod. no. qRnoCID0001262), PRKAA2/AMPKα2 (prod. no. qRnoCID0006799), ESR1/ERα (prod. no. qRnoCID0009588), ESR2/ERβ (prod. no. qRnoCID0008785), GPER (prod. no. qRnoCED0007818), Ghrh-R (prod. no. qRnoCED0003825), and the internal control gene GAPDH (prod. no. qRnoCID0057018; Bio-Rad) with SsoAdvanced^™^ PreAmp Supermix (prod. no. 1725160; Bio-Rad). Pre-amplified cDNA was generated by adding preamplification master mix (9.5 µL) to individual cDNA samples before thermal cycler incubation at 95 °C (3 min), followed by 18 cycles of incubation at 95 °C (15 sec), then 58 °C (4 min). Pre-amplified cDNA samples were diluted with IDTE (185 µL; prod. no. 11-05-01-05; 1X TE solution; Integrated DNA Technologies, Inc., Coralville, IA).

#### RT-qPCR Analysis

PCR samples were prepared by combining Bio-Rad primers [SF-1/NR5A1 (0.5 µL; prod. no. qRnoCID0001458), GAD1/GAD_67_ (0.5 µL; prod. no. qRnoCID0004554), GAD2/GAD_65_ (0.5 µL; prod. no. qRnoCID0003485), Ghrh (0.5 µL; prod. no. qRnoCID0007723), Ghrh-R (0.5 µL; prod. no. qRnoCED0003825, nNOS/NOS1 (0.5 µL; prod. no. qRnoCED0009301), GLS (0.5 µL; prod. no. qRnoCID0007756), PRKAA1/AMPKα1 (0.5 µL; prod. no. qRnoCID0001262), PRKAA2/AMPKα2 (0.5 µL; prod. no. qRnoCID0006799), ESR1 (0.5 µL; prod. no. qRnoCID0009588), ESR2 (0.5 µL; prod. no. qRnoCID0008785), GPER (0.5 µL; prod. no. qRnoCED0007818), and GAPDH (0.5 µL; prod. no. qRnoCID0057018)], cDNA sample (*2* µL), and iTaq^™^ Universal SYBR^®^ Green Supermix *(*5 µL, prod. no. 1725121*;* Bio-Rad). PCR samples were added to individual wells of hard-shell 384-well PCR plates (prod. no. HSP3805, Bio-Rad) for analysis in a CFX384^TM^ Touch Real-Time PCR Detection System (Bio-Rad) as follows: initial 30 sec 95 °C denaturation, followed by 40 cycles of (1) 3 sec incubation at 95 °C and (2) 45 sec incubation at 60 °C for GAD1/GAD_67_, ESR1; 60.5 °C for PRKAA1/AMPKα1, PRKAA2/AMPKα2; 59.9 °C for GAD2/GAD_65_, ESR2; 59.8 °C for GPER; 59.1 °C for SF-1/NR5A1; 58.8 °C for GLS; 58.5 °C for Ghrh; 58 °C for nNOS/NOS1; or 57.3 °C for GAPDH, respectively. Melt curve analyses were carried out to detect nonspecific products and primer dimers. Data were analyzed by the comparative Ct (2^-ΔΔCt^) method (Livak & Schmittgen, 2001).

### Micropunch Dissection of VMNdm SF-1 Tissue for Western Blot Analysis

For each animal, VMNdm tissue was bilaterally micropunch-dissected from 100 µm-thick frozen sections using calibrated hollow needle tools (Stoelting Co., Wood Dale, IL), and pooled in lysis buffer [2% sodium dodecyl sulfate (SDS), 0.05 M dithiothreitol, 10% glycerol, 1 mM EDTA, 60 mM Tris-HCl, pH 7.2]. Within each treatment group, VMNdm tissue lysate aliquots from individual subjects were combined to create triplicate sample pools for SF-1 protein immunoblot analysis. Sample pool proteins were separated by electrophoresis in Bio-Rad TGX 10% stain-free gels (prod. no. 1610183). Stain-Free imaging technology for total protein measurement was used as the loading control, as described (Bheemanapally et al., 2021; Briski et al., 2020; Ibrahim et al., 2020; Roy et al., 2023). After separation, gels were activated by UV light (1 min) in a Bio-Rad ChemiDoc MP Imaging System for quantification of total protein in each lane. Proteins were transferred to 0.45-μm PVDF-Plus membranes (prod. no. 121639; Data Support Co., Panorama City, CA), which were processed by FreedomRocker^™^ Blotbot^®^ (Next Advance, Inc., Troy, NY) automation. Membranes were blocked with either TBS containing 0.1% Tween-20 and 2% bovine serum albumin or SuperBlock^™^ Blocking Buffer (ThermoFisherSci.). Membranes were incubated (36-42 hr; 4 °C) with a rabbit anti-SF-1 primary antiserum (prod. no. PA5-41967, 1:2000; Invitrogen, Waltham, MA), then exposed to goat anti-rabbit horseradish peroxidase-labeled secondary antibodies (1:5000; prod. no. NEF812001EA; PerkinElmer, Waltham, MA), followed by the maximum sensitivity SuperSignal West Femto chemiluminescent substrate (prod. no. 34096; ThermoFisherSci.). Chemiluminescence optical density (O.D.) values for target protein bands were normalized to total in-lane protein using ChemiDoc MP Image Lab^™^ 6.0.0 software. Bio-Rad StainFree gel chemistry features incorporation of a proprietary trihalo compound that lacks inherent fluorescence, yet renders in-gel proteins fluorescent and thus measurable upon UV photoactivation. The formula used for normalization is the ratio of specific target protein O.D./total in-lane protein O.D. After summation of individual protein O.D. measures in a single lane, software relates the total protein O.D. value to target protein O.D., thereby deriving a normalized O.D. value. Each Western blot analysis employed precision plus protein molecular weight dual color standards (prod. no. 161-0374, Bio-Rad). Our figures depict mean normalized O.D. measures along the Y axis.

### Circulating Glucose and Counterregulatory Hormone Profiles

Plasma glucose concentrations were measured in duplicate for each subject using an ACCU-CHECK Aviva-plus glucometer (Roche Diagnostic Corporation, Indianapolis, IN), as described (Napit et al., 2019). Circulating corticosterone (prod. no. ADI-900-097; Enzo Life Sciences, Inc., Farmingdale, NY), glucagon (prod. no. EZGLU-30K, EMD Millipore, Billerica, MA), and GH (prod. no. KRC5311; Invitrogen, Waltham, MA] concentrations were determined in duplicate using commercial ELISA kit reagents, as reported (Ibrahim et al., 2019; Sapkota et al., 2023).

### Statistics

Mean normalized mRNA, normalized SF-1 protein, glucose, and counterregulatory hormone values were analyzed among treatment groups by two-way analysis of variance and Student Newman Keuls *post-hoc* test. Differences of *p* < 0.05 were considered significant. In each figure, statistical differences between specific pairs of treatment groups are denoted as follows: *p < 0.05; **p < 0.01; ***p < 0.001.

## Results

VMNdm Ghrh neurons express the transcription factor SF-1, which exerts well-documented regulatory effects on energy and glucose homeostasis. Current research employed siRNA reagents to repress VMN SF-1 gene expression *in vivo* to address the premise that in female rat Ghrh neurons, SF-1 controls eu- and/or hypoglycemia-associated expression patterns of gene transcripts that generate counterregulatory neurotransmitter biosynthetic pathway, AMPK catalytic subunit, and ER variant proteins. Individual VMNdm Ghrh neurons were obtained by *in situ* gene immunocytochemistry/laser-catapult-microdissection methods for single-cell multiplex single-cell qPCR analyses. Present work also evaluated whether VMN SF-1 gene expression is critical for optimal eu- and/or hypoglycemic patterns of corticosterone, glucagon, and GH secretion in this sex.

**Figure 1.**
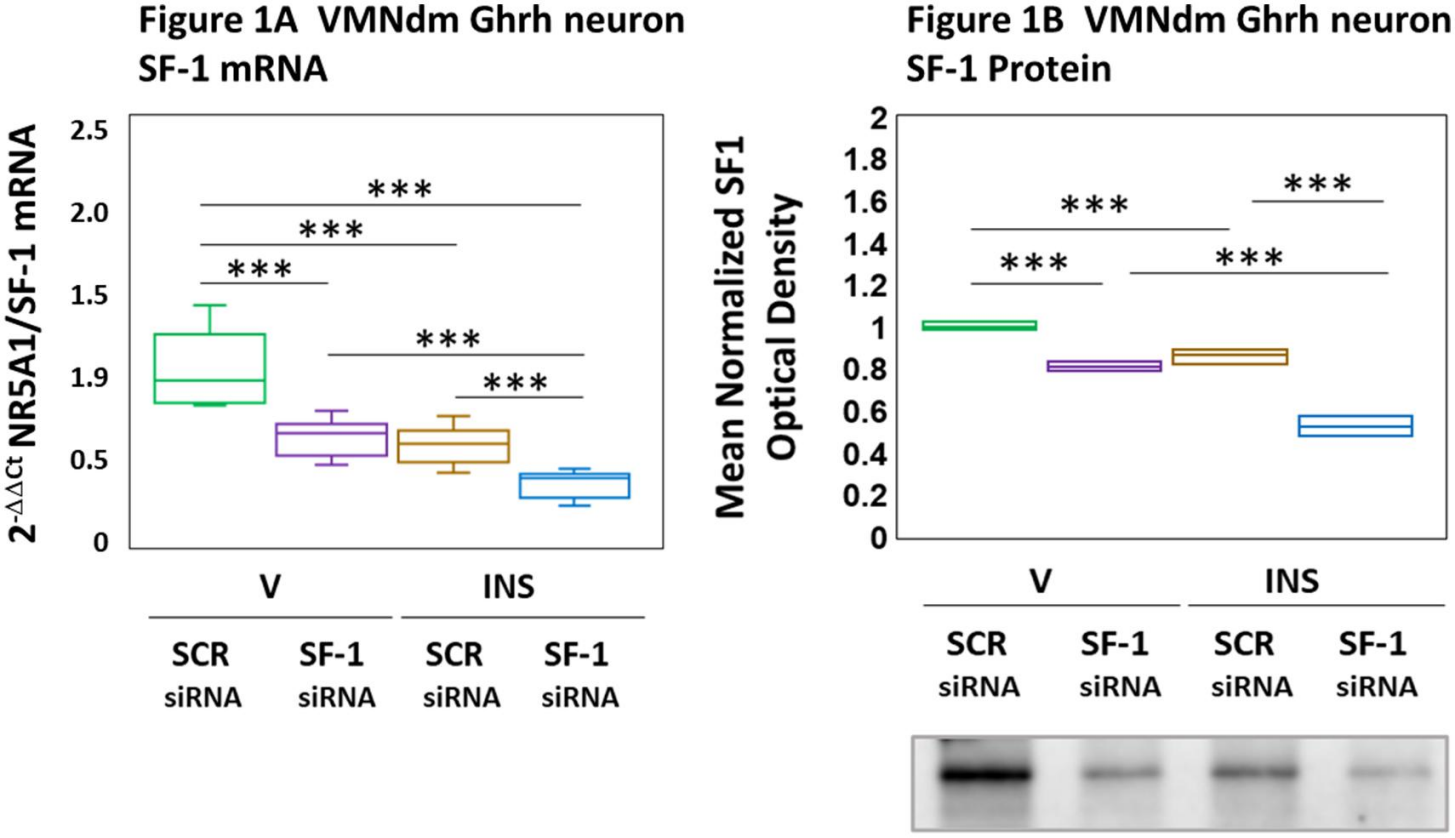
Ventromedial Hypothalamic Nucleus Steroidogenic Factor-1 (SF-1) Gene Knockdown Effects on Dorsomedial VMN (VMNdm) Growth Hormone Releasing Hormone (Ghrh) Neuron SF-1 mRNA and VMNdm SF-1 Protein Profiles in Eu- and Hypoglycemic Female Rats. After random assignment to one of 4 treatment groups (*n* = 6/group), female rats were pretreated by bilateral intra-VMN delivery of control/scramble (SCR) or SF-1 siRNA 7 days before subcutaneous (sc) vehicle (V) or neutral protamine Hagedorn insulin (INS; 10.0 U/kg *bw*) injection. Individual brains were cut into alternating 10 or 100 µm-thick fresh frozen sections for single-cell Ghrh-immunopositive neuron laser-catapult-microdissection or VMNdm tissue micropunch-dissection, respectively. Figure A depicts single-cell qPCR SF-1 mRNA analysis results. Data are presented in box-and-whisker plot format, which displays the median, lower and upper quartiles, and lower and upper extremes of a data set. Plots depict mean normalized VMNdm Ghrh neuron SF-1 transcript measures for the following treatment groups: SCR siRNA/V (green box-and-whisker plots, *n* = 12); SF-1 siRNA/V (purple box-and-whisker plots; *n* = 12); SCR siRNA/INS (brown box-and-whisker plots; *n* = 12; SF-1 siRNA/INS (blue box-and-whisker plots; *n* = 12). For each treatment group, aliquots of micropunched VMNdm tissue obtained from each animal were pooled to generate triplicate sample pools for SF-1 protein Western blot analysis. Figure B depicts mean SF-1 protein optical density (O.D.) measures for treatment groups described above. Normalized mRNA and protein data were analyzed by two-way ANOVA and Student-Newman-Keuls *post-hoc* test, using GraphPad Prism, Vol. 8 software. Statistical differences between discrete pairs of treatment groups are denoted as follows: *p < 0.05; **p < 0.01; ***p < 0.001.

Data shown in Figure represent VMN SF-1 gene knockdown effects on VMNdm Ghrh nerve cell SF-1 (Figure 1A) and protein (Figure 1B) expression in eu- or hypoglycemic female rats. Results portrayed in Figure 1A indicate that SF-1 siRNA administration to the VMN significantly repressed SF-1 mRNA levels in Ghrh neurons acquired from vehicle-injected controls [F_(3,44)_ = 48.74, *p* < 0.001; Pretreatment effect: F_(1.44)_ = 62.34, *p* < 0.001; Treatment effect: F_(1,44)_ = 79.89, *p* < 0.001; Pretreatment/treatment interaction: F_(1,44)_ = 3.99, *p* = 0.052]. Hypoglycemia was inhibitory to this gene profile; SF-1 siRNA pretreatment exacerbated this negativeresponse. Results presented in Figure 1B show that VMN SF-1 gene silencing decreased VMNdm SF-1 tissue protein content in the female rat [F_(3,8)_ = 115.17, *p* < 0.001; Pretreatment effect: F_(1.8)_ = 199.25, *p* < 0.001; Treatment effect: F_(1,8)_ = 131.75, *p* < 0.001; Pretreatment/treatment interaction: F_(1,8)_ = 14.50, *p* = 0.005]. Data show that microdissected VMNdm tissue exhibited hypoglycemia-associated reductions in VMNdm SF-1 protein; SF-1 siRNA pretreatment amplified this decline in protein expression. Results confirm efficacy of the current gene knockdown treatment paradigm for inhibition of VMNdm Ghrh SF-1 gene expression and down-regulation of VMNdm SF-1 protein content.

**Figure 2.**
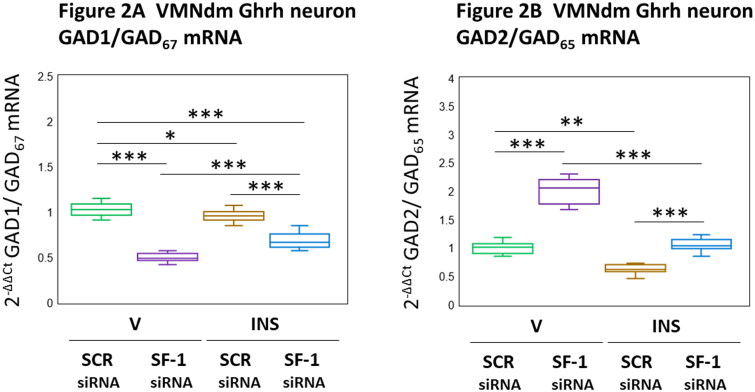
Eu- versus Hypoglycemic Patterns of VMNdm Ghrh Neuron Glutamate Decarboxylase (GAD)_67_/GAD1 and GAD_65_/GAD2 Gene Expression in the Female Rat: Impact of VMN SF-1 Gene Silencing. Results depict mean normalized GAD1 (Figure 2A) and GAD2 (Figure 2B) mRNA profiles for VMNdm Ghrh neurons laser-microdissected from female subjects that were treated as follows: SCR siRNA/V (green box-and-whisker plots; *n* = 12); SF-1 siRNA/V (purple box-and-whisker plots; *n* = 12); SCR siRNA/INS (brown box-and-whisker plots; *n* = 12); SF-1 siRNA/INS (blue box-and-whisker plots; *n* = 12). Normalized mRNA data were analyzed by two-way ANOVA and Student-Newman-Keuls *post-hoc* test, using GraphPad Prism, Vol. 8 software. Statistical differences between discrete pairs of treatment groups are denoted as follows: *p < 0.05; **p < 0.01; ***p < 0.001.

The amino acid neurotransmitter GABA imposes a suppressive tone on counterregulatory hormone secretion. The rate-limiting enzyme GAD is expressed in brain as GAD1/GAD_67_ and GAD2/GAD_65_ kDa molecular weight isoforms that exhibit dissimilar amino acid primary structure, nerve cell subcellular localization, and regulation (Behar, 2009). The distinguishing mRNAs that encode these GAD variants are co-expressed in VMNdm Ghrh neurons in both sexes. Figure 2 illustrates effects of VMN SF-1 siRNA administration on eu- and hypoglycemic patterns of VMNdm Ghrh neuron GAD1 (Figure 2A [F_(3,44)_ = 133.11, *p* < 0.001; Pretreatment effect: F_(1.44)_ = 356.43, *p* < 0.001; Treatment effect: F_(1,44)_ = 7.06, *p* = 0.011; Pretreatment/treatment interaction: F_(1,44)_ = 35.82, *p* < 0.001]) and GAD2 (Figure 2B [F_(3,44)_ = 210.40, *p* < 0.001; Pretreatment effect: F_(1.44)_ = 310.79, *p* < 0.001; Treatment effect: F_(1,44)_ = 271.61, *p* < 0.001; Pretreatment/treatment interaction: F_(1,44)_ = 48.80, *p* < 0.001]) gene expression. SF-1 gene knockdown elicited divergent adjustments in baseline GAD1 and GAD2 transcription in this neuron population, causing down- or up-regulation of these mRNA profiles, respectively. VMNdm Ghrh neurons exhibited reductions in both GAD transcripts during hypoglycemia. SF-1 siRNA pretreatment had opposite effects on hypoglycemic patterns of GAD1 versus GAD2 mRNA expression. Current data show that SF-1 gene silencing aggravated hypoglycemic inhibition of GAD1 gene expression, yet prevented reductions in GAD2 transcription in VMNdm Ghrh neurons. Results show that SF-1 imposes divergent control of baseline and hypoglycemic patterns of VMNdm Ghrh GAD1 (stimulatory) and GAD2 (inhibitory) gene expression in the female rat.

**Figure 3.**
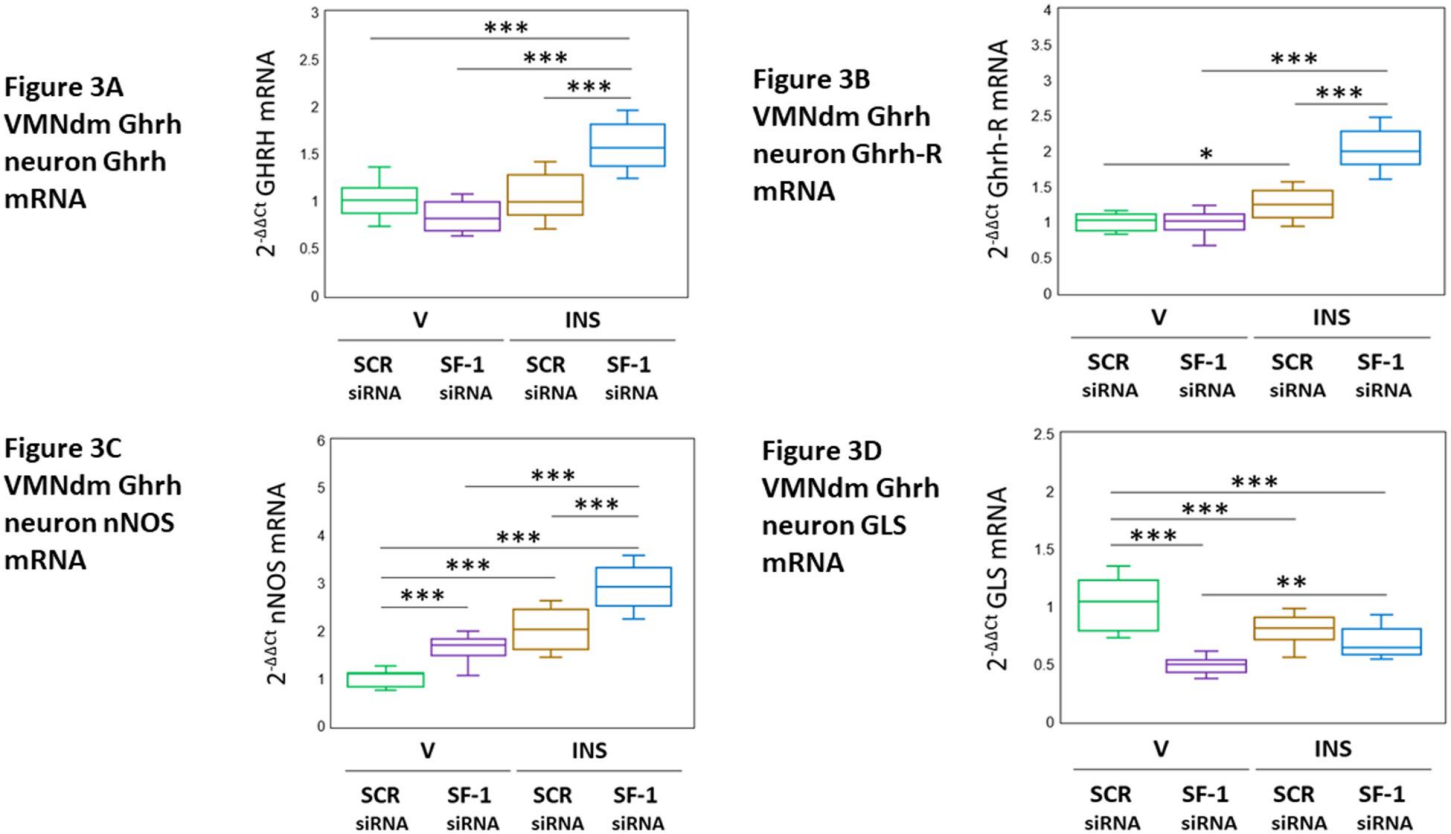
Effects ofVMN SF-1 Gene Silencing on Hypoglycemic Patterns of Growth Hormone-Releasing Hormone (Ghrh), Ghrh Receptor (Ghrh-R), Nitric Oxide Synthase (nNOS), and Glutaminase (GLS) mRNA Expression in Female Rat VMNdm Ghrh Neurons. Data show mean normalized Ghrh (Figure 3A), Ghrh-R (Figure 3B), nNOS (Figure 3C), and GLS mRNA measures for VMNdm Ghrh neurons collected by laser-microdissection after SCR siRNA/V (*n* = 12); SF-1 siRNA/V (*n* = 12); SCR siRNA/INS (*n* = 12); or SF-1 siRNA/INS (*n* = 12) treatment. Normalized mRNA data were analyzed by two-way ANOVA and Student-Newman-Keuls *post-hoc* test, using GraphPad Prism, Vol. 8 software. Statistical differences between discrete pairs of treatment groups are denoted as follows: *p < 0.05; **p < 0.01; ***p < 0.001.

VMNdm Ghrh neurons produce several neurochemicals that stimulate counterregulatory hormone release, namely the neuropeptide neurotransmitter Ghrh, the freely-diffusable gas NO, and the amino acid transmitter glutamate. Data presented in Figure 3 depict VMN SF-1 gene knockdown effects on expression profiles of gene that encode Ghrh (Figure 3A [F_(3,44)_ = 27.28, *p* < 0.001; Pretreatment effect: F_(1.44)_ = 7.96, *p* = 0.0071; Treatment effect: F_(1,44)_ = 39.66, *p* < 0.001; Pretreatment/treatment interaction: F_(1,44)_ = 34.20, *p* < 0.001]) or the neurochemical biosynthetic enzyme markers nNOS (Figure 3C [F_(3,44)_ = 63.99, *p* < 0.001; Pretreatment effect: F_(1.44)_ = 60.67, *p* < 0.001; Treatment effect: F_(1,44)_ = 129.08, *p* < 0.001; Pretreatment/treatment interaction: F_(1,44)_ = 2.22, *p* = 0.143]) and GLS (Figure 3D [F_(3,44)_ = 27.08, *p* < 0.001; Pretreatment effect: F_(1.44)_ = 56.88, *p* < 0.001; Treatment effect: F_(1,44)_ = 0.17, *p* = 0.679; Pretreatment/treatment interaction: F_(1,44)_ = 24.19, *p* < 0.001]). As shown in Figure 3A, SF-1 gene silencing did not modify Ghrh gene transcription in euglycemic female rats, but enhanced this gene profile in hypoglycemic animals. Previous studies showed that Ghrh regulates co-expressed neurotransmitter marker mRNA profiles in VMNdm Ghrh neurons, and infer this control may involve Ghrh-R. Data presented in Figure 3B indicate that euglycemic patterns of VMNdm Ghrh neuron Ghrh-R gene expression are refractory to SF-1 mRNA, whereas this siRNA pretreatment augmented Ghrh-R mRNA levels in cell samples obtained from hypoglycemic rats [F_(3,44)_ = 66.31, *p* < 0.001; Pretreatment effect: F_(1.44)_ = 40.22, *p* < 0.001; Treatment effect: F_(1,44)_ = 112.16, *p* < 0.001; Pretreatment/treatment interaction: F_(1,44)_ = 46.57, *p* < 0.001]. As shown in Figure 3C, nNOS mRNA levels in female rat VMNdm Ghrh neuron were elevated in response to SF-1 gene knockdown. Hypoglycemia-associated up-regulation of this gene profile was amplified by SF-1 siRNA pretreatment. Results presented in Figure 3D reveal that SF-1 gene knockdown suppressed GLS mRNA levels in this neuron population, yet had no effect on hypoglycemic suppression of GLS gene expression. Outcomes show that hypoglycemia elicits a gain of SF-1 inhibitory control of Ghrh and Ghrh-R mRNA profiles as well as loss of stimulatory influence on GLS transcription in VMNdm Ghrh neurons in the female rat. Data also disclose a positive SF-1 impact on eu- and hypoglycemic patterns of nNOS gene expression.

**Figure 4.**
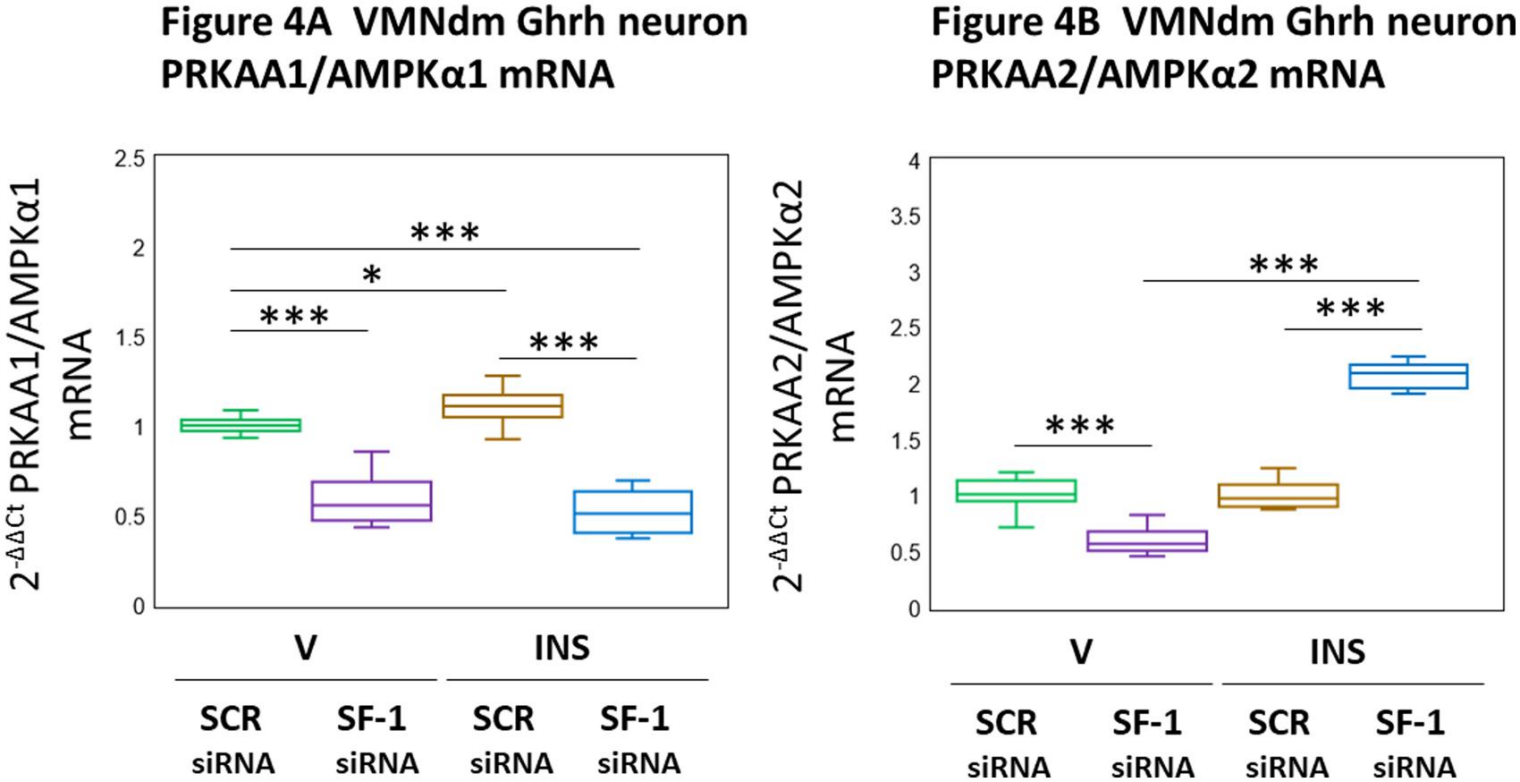
Effects of VMN SF-1 siRNA Pretreatment on Hypoglycemic Patterns of VMNdm Ghrh Neuron PRKAA1/AMPKα1 and PRKAA2/AMPKα2 Gene Expression in the Female rat. Data depict mean normalized PRKAA1/AMPKα1 (Figure 4A) and PRKAA2/AMPKα2 (Figure 4B) mRNA values for VMNdm Ghrh neurons collected after SCR siRNA/V (*n* = 12); SF-1 siRNA/V (*n* = 12); SCR siRNA/INS (*n* = 12); or SF-1 siRNA/INS (*n* = 12) treatment. Normalized mRNA data were analyzed by two-way ANOVA and Student-Newman-Keuls *post-hoc* test, using GraphPad Prism, Vol. 8 software. Statistical differences between discrete pairs of treatment groups are denoted as follows: *p < 0.05; **p < 0.01; ***p < 0.001.

Data shown in Figure 4 illustrate SF-1 gene silencing effects on female rat VMNdm Ghrh neuron PRKAA1/AMPKα1 (Figure 4A [F_(3,44)_ = 92.92, *p* < 0.001; Pretreatment effect: F_(1.44)_ = 270.08, *p* < 0.001; Treatment effect: F_(1,44)_ = 0.21, *p* = 0.652; Pretreatment/treatment interaction: F_(1,44)_ = 8.49, *p* = 0.006]) and PRKAA2/AMPKα2 (Figure 4B [F_(3,44)_ = 299.92, *p* < 0.001; Pretreatment effect: F_(1.44)_ = 81.05, *p* < 0.001; Treatment effect: F_(1,44)_ = 403.42, *p* < 0.001; Pretreatment/treatment interaction: F_(1,44)_ = 415.27, *p* < 0.001]) gene transcription. Data show that VMN SF-1 gene knockdown diminished PRKAA1/AMPKα1 and PRKAA2/AMPKα2 mRNAs in this nerve cell population. Hypoglycemia caused dissimilar changes in these gene profiles, as INS-injected animals showed enhanced PRKAA1/AMPKα1 gene expression, yet unaltered PRKAA2/AMPKα2 mRNA profiles. SF-1 siRNA pretreatment reversed hypoglycemic stimulation of PRKAA1/AMPKα1 mRNA, but amplified hypoglycemic patterns of PRKAA2/AMPKα2 gene transcription. These results infer that SF-1 is stimulatory to baseline expression patterns of VMNdm Ghrh neuron PRKAA1/AMPKα1 and PRKAA2/AMPKα2 gene expression, yet exerts divergent control of these 2 mRNA profiles during hypoglycemia, namely stimulation of PRKAA1/AMPKα1 alongside inhibition of PRKAA2/AMPKα2.

**Figure 5.**
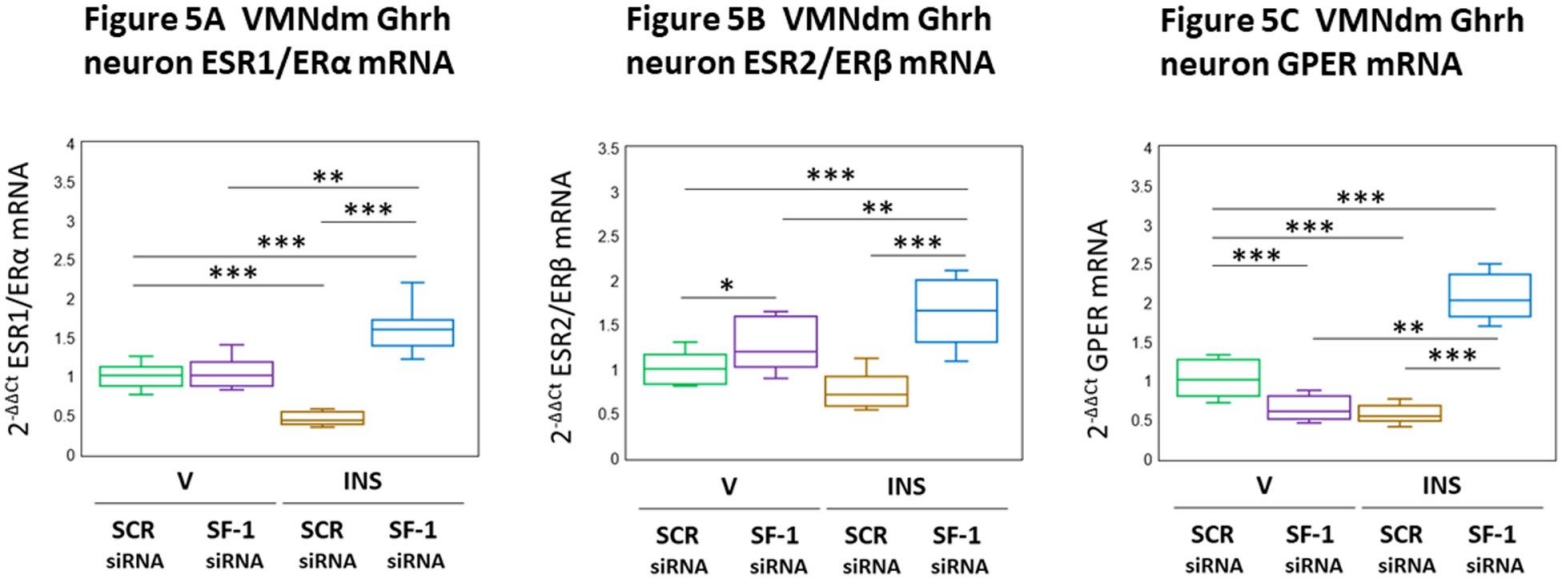
Effects of VMN SF-1 Gene Silencing on Patterns of VMNdm Ghrh Neuron Estrogen Receptor (ER)-Alpha (ESR1/ERα), ER-Beta (ESR2/ERβ), and G Protein-Coupled Membrane Estrogen Receptor-1 (GPER) Gene Expression in V- or INS-Injected Female Rats. Data depict mean normalized ESR1 (Figure 5A), ESR2 (Figure 5B), and GPER (Figure 5C) gene expression in VMNdm Ghrh neurons after the following combinatory treatments: SCR siRNA/V (*n* = 12); SF-1 siRNA/V (*n* = 12); SCR siRNA/INS (*n* = 12); SF-1 siRNA/INS (*n* = 12). Normalized mRNA data were analyzed by two-way ANOVA and Student-Newman-Keuls *post-hoc* test, using GraphPad Prism, Vol. 8 software. Statistical differences between discrete pairs of treatment groups are denoted as follows: *p < 0.05; **p < 0.01; ***p < 0.001.

Results depicted in Figure 5 illustrate effects of VMN SF-1 gene knockdown on VMNdm Ghrh nerve cell nuclear [ESR1/ERα (Figure 5A [F_(3,44)_ = 77.26, *p* < 0.001; Pretreatment effect: F_(1.44)_ = 123.76, *p* < 0.001; Treatment effect: F_(1,44)_ = 0.004, *p* = 0.952; Pretreatment/treatment interaction: F_(1,44)_ = 108.00, *p* < 0.001)]; ESR1/ERβ (Figure 5B [F_(3,44)_ = 24.74, *p* < 0.001; Pretreatment effect: F_(1.44)_ = 57.41, *p* < 0.001; Treatment effect: F_(1,44)_ = 0.51, *p* = 0.478; Pretreatment/treatment interaction: F_(1,44)_ = 16.30, *p* < 0.001)] and membrane [GPER (Figure 5C [F_(3,44)_ = 135.34, *p* < 0.001; Pretreatment effect: F_(1.44)_ = 88.60, *p* < 0.001; Treatment effect: F_(1,44)_ = 66.34, *p* < 0.001; Pretreatment/treatment interaction: F_(1,44)_ = 251.09, *p* < 0.001)] gene expression. Results show that SF-1 gene silencing increased or decreased baseline ESR2 and GPER mRNA profiles, respectively, but did not alter ESR1 transcription. Hypoglycemia decreased VMNdm Ghrh nerve cell ESR1 and GPER gene expression; ESR2 mRNA levels were unaffected by this metabolic challenge. SF-1 siRNA pretreatment increased transcription of each ER variant in hypoglycemic female rats.

**Figure 6.**
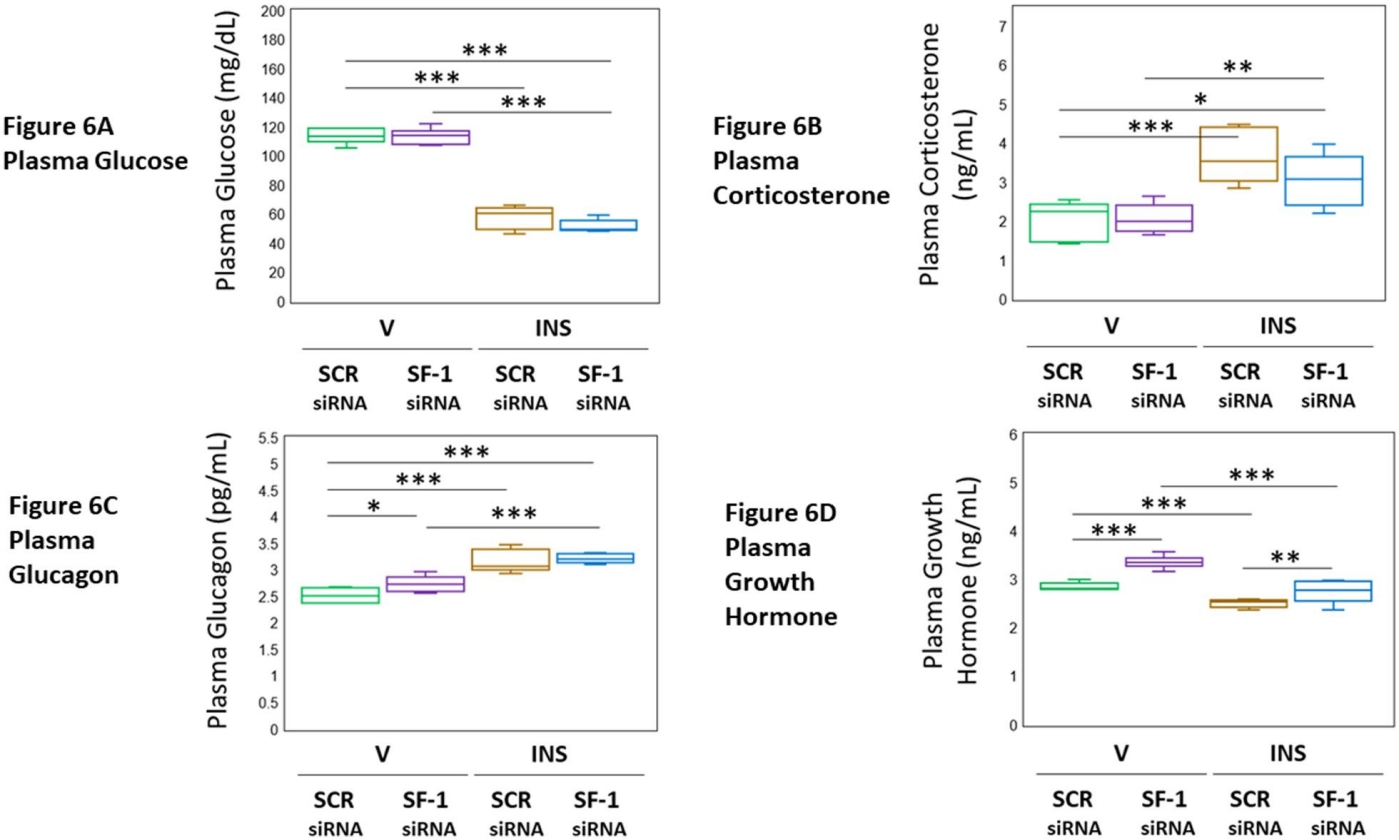
Effects of VMN SF-1 Gene Knockdown on Plasma Glucose and Counterregulatory Hormone Profiles in Eu- or Hypoglycemic Female rats. Plasma samples were obtained from groups of SCR or SF-1 siRNA-pretreated male rats one hour after *sc* injection of V or INS, and analyzed for glucose (Figure 6A), glucagon (Figure 6B), corticosterone (Figure 6C), or growth hormone (Figure 6D) concentrations. In each panel, individual treatment group data depict mean plasma concentrations ± S.E.M. for *n* = 6 samples. Data were analyzed by two-way ANOVA and Student-Newman-Keuls *post-hoc* test, using GraphPad Prism, Vol. 8 software. *p < 0.05, **p < 0.01, ***p < 0.001.

Figure 6 presents VMN SF-1 gene silencing effects on circulating glucose and counterregulatory hormone levels in female rats injected with vehicle or INS. Data presented in Figure 6A show that this genetic manipulation did not affect eu- or hypoglycemia in this sex (F_(3,20)_ = 200.69, *p* < 0.001; Pretreatment effect: F_(1.20)_ = 1.74, *p* = 0.203; Treatment effect: F_(1,20)_ = 598.79, *p* < 0.001; Pretreatment/treatment interaction: F_(1,20)_ = 1.56, *p* = 0.227). Baseline and hypoglycemic patterns of corticosterone [Figure 6B (F_(3,20)_ = 11.03, *p* < 0.001; Pretreatment effect: F_(1.20)_ = 1.58, *p* = 0.223; Treatment effect: F_(1,20)_ = 29.90, *p* < 0.001; Pretreatment/treatment interaction: F_(1,20)_ = 1.61, *p* = 0.219)] were refractory to VMN SF-1 siRNA administration. As shown in Figure 6C, baseline glucagon secretion profiles were elevated in response to SF-1 gene knockdown (F_(3,20)_ = 11.03, *p* < 0.001; Pretreatment effect: F_(1.20)_ = 1.58, *p* = 0.223; Treatment effect: F_(1,20)_ = 29.90, *p* < 0.001; Pretreatment/treatment interaction: F_(1,20)_ = 1.61, *p* = 0.219), but this genetic manipulation had no effect on hypoglycemic hyperglucagonemia. Results presented in Figure 6D indicate that basal and hypoglycemic patterns of GH release were augmented by SF-1 gene knockdown (F_(3,20)_ = 37.74, *p* < 0.001; Pretreatment effect: F_(1.20)_ = 39.77, *p* < 0.001; Treatment effect: F_(1,20)_ = 68.32, *p* < 0.001; Pretreatment/treatment interaction: F_(1,20)_ = 5.14, *p* = 0035). Outcomes show that in the female rat, VMN SF-1 does not affect circulating glucose profiles in V- or INS-injected animals or corticosterone or glucagon secretory responses to hypoglycemia. However, this transcription factor evidently acts within the VMN to direct hypoglycemic suppression of pituitary GH outflow in this sex.

## Discussion

The transcription factor SF-1 acts within the brain to regulate metabolic homeostasis (Choi et al., 2013; Garfield et al., 2014; Kim et al., 2011; Meek et al., 2016; Xu et al., 2011; Zhang et al., 2008). VMNdm Ghrh neurons in each sex express SF-1 alongside chemically-diverse neurotransmitters that shape glucose counterregulation (Sapkota et al., 2023). Recent studies show that in male rats, SF-1 controls neurotransmitter marker, energy sensor, and ER variant gene expression in that distinctive neuron population (Sapkota et al., 2024). Current research extends those findings by addressing the premise that SF-1 may impose sex-specific control of glucose-regulatory signaling, AMPK catalytic function, and estradiol receptivity in VMNdm Ghrhs population during eu- and hypoglycemia. Results show that in the female rat, VMN SF-1 gene silencing suppressed or enhanced glutamate decarboxylase (GAD)-1 (GAD1/GAD67) or -2 (GAD2/GAD65) transcription, respectively, after V or INS injection. This genetic manipulation also up-regulated nNOS mRNA profiles under eu- and hypoglycemia. Hypoglycemia caused a corresponding gain or loss of SF-1 control of Ghrh and GLS gene transcription. SF-1 gene knockdown had divergent effects on hypoglycemic expression patterns of energy sensor 5’-AMP-activated protein kinase (AMPK)-alpha1 and -alpha2 mRNAs. SF-1 regulation of ERα and GPER gene profiles was observed to vary according to glucose status, but was inhibitory to ERβ transcription in both V and INS treatment groups. In the female, SF-1 knockdown attenuated GH, but not corticosterone or glucagon secretory responses to hypoglylcemia. Results show that SF-1 expression is critical for female rat VMNdm Ghrh neuron counterregulatory neurochemical, AMPK catalytic subunit, and ER variant gene transcription responses to hypoglycemia. Sex differences in direction of SF-1 control of distinctive glucose-regulatory transmitter gene profiles may underlie observed disparities in SF-1 regulation of counterregulatory hormone secretion in the two sexes. In male, hypoglycemic patterns of corticosterone and glucagon secretion may be governed principally by SF-1 sensitive neural pathways, whereas this endocrine outflow may be governed by parallel, redundant SF-1-dependent and -independent mechanisms in female.

Results verify the efficacy of the current gene knockdown treatment paradigm for parallel down-regulation of VMNdm Ghrh nerve cell SF-1 gene expression and VMNdm SF-1 protein. While it is reasonable to presume that this treatment reduces Ghrh neuron SF-1 protein levels in eu- and hypoglycemic female rats, experimental confirmation of this outcome will require application of analytical methods of requisite sensitivity for protein quantification in single brain cell samples. The possibility that SF-1 gene knockdown may affect the functional status of other SF-1-expressing VMNdm neurotransmitter neuron populations should not be dismissed. Current data infer that, in the female, SF-1 regulation of VMNdm Ghrh nerve cell Ghrh and Ghrh-R gene transcription is contingent upon glucose status as SF-1 siRNA administration did not affect these gene profiles in euglycemic subjects, but augmented both transcripts in INS-injected animals. The mechanisms that underlie this evident hypoglycemia-associated gain in SF-1 inhibitory control remain unclear. As Ghrh and Ghrh-R mRNA levels were unaffected by hypoglycemia in this sex, it is likely that a SF-1-mediated suppressive tone is counterbalanced by positive stimuli. Measurable adjustments in these gene profiles should not be considered definitive proof of corresponding changes in neuropeptide or neuropeptide receptor protein production. Interestingly, outcomes of studies in the male document SF-1 stimulation of baseline and hypoglycemic patterns of Ghrh and Ghrh-R gene expression (Table 1). A critical issue that warrants further investigation is the lack of insight on potential factors that determine directionality of SF-1 control of Ghrh signaling by VMNdm Ghrh neurons in each sex.

**Table 1.** Steroidogenic factor-1 (SF-1) regulation of ventromedial hypothalamic nucleus growth hormone-releasing hormone (Ghrh) neuron transmitter marker, energy sensor, and estrogen receptor (ER) variant gene expression in female versus male rats.

	Male Ghrh neurons			Female Ghrh neurons		
	**SF-1 KD** ^a^	**IIH** ^b^	SF-1/IIH	SF-1 KD	IIH	SF-1KD/IH
Ghrh	↓^c^	N.E.^d^	↓	N.E.	N.E.	↑^e^
Ghrh-R^f^	↓	↑	↓	N.E.	↑	↑
GAD1/GAD_67_^g^	↓	↓	↓	↓	↓	↓
GAD2/GAD_65_^h^	↓	↓	↑	↑	↓	↑
nNOS^i^	↓	↑	↓	↑	↑	↑
GLS^j^	↑	↓	↑	↓	↓	↓
PRKAA1^k^	↑	↑	↓	↓	↑	↓
PRKAA2^l^	↑	↑	↓	↓	N.E.	↑
ESR1^m^	↓	↓	↓	N.E.	↓	↑
ESR2^n^	↑	↓	↑	↑	N.E.	↑
GPER^o^	↓	↓	↓	↓	↓	↑
Glucagon	↑	↑	↑	↑	↑	N.E.
Corticosterone	↓	↑	↑	N.E.	↑	N.E.
GH	↑	↑	↓	↑	↓	↑

^a^ Knockdown.

^b^ Insulin-induced hypoglycemia; 10.0 U neutral protamine Hagedorn insulin/kg *bw sc*.

^c^ Decreased expression.

^d^ No effect.

^e^ Increased expression.

^f^ Ghrh-receptor.

^g^ Glutamate decarboxyase_67_.

^h^Glutamate decarboxylase_65_.

^i^ neuronal nitric oxide synthase.

^j^ glutaminase.

^k^ 5’-AMP-activated protein kinase (AMPK) alpha subunit-1.

^l^ AMPK alpha subunit-2.

^m^ Estrogen receptor-alpha.

^n^Estrogen receptor-beta.

^o^ G protein-coupled estrogen receptor-1.

The amino acid neurotransmitter GABA imposes a negative tone on glucose counterregulation (Chan et al., 2006, 2008). GABA biosynthesis involves the rate-limiting enzyme GAD, which exists as 67 kDa GAD1/GAD_67_ and 65 kDa GAD2/GAD_65_ size variants encoded by distinctive genes. GAD isoforms exhibit dissimilar amino acid primary structure, neuronal subcellular distribution, and regulation. VMNdm Ghrh neurons express GAD1 and GAD2 transcripts (Sapkota et al., 2023). GAD1 expression is subject to transcriptional and posttranscriptional control, whereas GAD2 is regulated by transcriptional and kinetic mechanisms (Behar, 2009). GAD2 is present in axon terminals and vesicles whereas GAD1 is localized to the cytoplasm, suggesting that distinctive vesicular versus cytoplasmic GABA pools function in relation to neurotransmission or cellular metabolic functions, respectively (Martin & Barke, 1998; Schousboe & Waagepetersen, 2017; Tavazzani et al., 2014). Current results show that in the female SF-1 imposes divergent control of GAD1 versus GAD2 gene expression, as the former profile was decreased by SF-1 gene knockdown in V- or INS-injected animals, whereas the latter was up-regulated by SF-1 siRNA in both treatment groups. In female, SF-1 may likely exert discriminatory command of vesicular versus cytoplasmic GABA production, including a negative brake on GABA neurotransmission. A notable sex difference involves evident opposite regulatory effects of SF-1 on baseline GAD2 mRNA profiles in male versus female, as this transcription factor up-regulates these transcripts in the former sex while inhibiting this gene profile in the latter (Table 1).

Previous work showed that VMNdm Ghrh neurons express glutaminase (GLS) (Sapkota et al., 2023), a critical enzyme component of the biosynthetic pathway that produces the counterregulation-enhancing amino acid neurotransmitter glutamate (Tong et al., 2007). Data here show that in the female SF-1 may augment baseline Ghrh nerve cell GLS gene expression, but this input is abolished during hypoglycemia. These findings differ from studies in the male, which disclose SF-1 inhibition or stimulation of GLS mRNA profiles in this neuron population during eu- or hypoglycemia, respectively (Table 1). Ghrh neurons also express the labile gas NO, which stimulates counterregulatory hormone outflow (Faber et al., 2018; Fioramonti et al., 2011). Current results show that SF-1 inhibits expression of mRNA encoding the marker enzyme nNOS in V- and INS-injected female animals, suggesting that this transcription factor may suppress NO release by Ghrh neurons. These findings deviate from outcomes of male studies, which document SF-1 stimulation of nNOS gene transcription during eu- and hypoglycemia. There remains a need to characterize the neurochemical phenotype and neuroanatomical location of cellular targets of Ghrh nerve cell Ghrh, GABA, glutamate, and NO signaling, and, furthermore, to establish how SF-1—controlled transmission patterns of these distinctive neurochemicals may shape downstream brain glucostatic network function.

The presence of energy sensor bio-machinery, namely the ultra-sensitive energy gauge AMPK, in VMNdm Ghrh neurons implies that they function as an interoceptive monitor of brain cell energy state. These neurons presumably supply this critical sensory information to the glucostatic network; it remains unclear which, if any of the hypoglycemia-sensitive co-expressed neurochemicals investigated here convey this input. It remains to be determined if AMPK activity state controls production and release of all or a subset of co-expressed transmitters generated in VMNdm Ghrh nerve cells. Current data show that in the female rat SF-1 up-regulates basal expression profiles of genes that encode PRKAA1 and PRKAA2 catalytic subunits, yet exerts divergent control of these transcripts during hypoglycemia, causing augmentation of the former, yet imposing an inhibitory tone on the latter under those conditions. These two catalytic subunit isoforms are activated to a similar extent in response to increases in intracellular AMP, but exhibit dissimilar substrate specificity. Evidence reported here shows that glucose status is a critical determinant of the direction of SF-1 control, e.g. stimulation versus inhibition of PRKAA2 gene expression in VMNdm Ghrh neurons. Further research is warranted to characterize mechanisms that achieve this evident glucose-dependent shift from positive to negative SF-1 tone on AMPKα2 mRNA expression. Differential glucose-dependent SF-1 regulation of PRKAA2 gene transcription rates may mediate adaptive adjustments in cell function in response to decrements in glucose supply. Unlike current results, outcomes from the male study show that SF-1 inhibits basal expression profiles of both PRKAA mRNAs in Ghrh neurons (Table 1). Another apparent sex difference involves SF-1 stimulation versus inhibition of hypoglycemic patterns of PRKAA2 gene expression in male and female rats, respectively. While it is intriguing to consider that AMPK may mediate SF-1 control of Ghrh neuron release of one or more glucose-regulatory neurochemicals, it should be noted that current studies did not determine if SF-1 knockdown affects the phosphorylation state of either PRKAA variant protein.

VMNdm Ghrh neurons are directly sensitive to estradiol by virtue of expression of multiple ER variant mRNAs, namely those that encode the nuclear receptors ERα and ERβ and membrane ER GPER (Sapkota et al., 2023). Present work shows that in female rats SF-1 regulates basal transcription of ERβ and GPER in these neurons, as SF-1 knockdown, respectively, augmented or inhibited these gene profiles. SF-1 evidently imposes sex-specific control of baseline ERα expression in VMNdm Ghrh neurons as silencing either had no effect (female) or suppressed (male) this gene profile. Present data align with prior reports that hypoglycemia down-regulates ERα and GPER gene profiles in female VMNdm Ghrh neurons, whereas ERβ transcription is unaffected (Sapkota et al., 2023). In female, hypoglycemia elicits a gain of negative SF-1 regulation of ERα mRNA in this cell population, whereas in the male, SF-1 regulation of this gene profile is stimulatory irrespective of normal versus lowered circulating glucose levels. The female also exhibits a switch in direction of SF-1 control of GPER, i.e. stimulatory-to-inhibitory, due to hypoglycemia; SF-1 regulation of this gene profile in the male is negative. The mechanisms that underlie these glucose-dependent changes in SF-1 regulation of VMNdm Ghrh neuron receptivity to estradiol in the female are not known. Collectively, the above findings infer that in the female, SF-1 may drive hypoglycemic suppression of ERα and GPER gene expression, yet may impose an opposite effect, e.g. blunting of this inhibitory effect in the male. There remains a critical need for insight on how SF-1—dependent adjustments in ER variant-controlled estradiol input to VMNdm Ghrh neurons during hypoglycemia may contribute to transmitter protein marker gene responses to that metabolic challenge.

The current research strategy involving *in vivo* SF-1 gene knockdown tools implicates this VMN transcription factor in neural control counterregulatory hormone outflow in the female rat. Present data show that VMN SF-1 gene silencing significantly enhances baseline glucagon and GH hormone secretion in the female rat, results that mirror outcomes of studies on the male. SF-1 regulation of euglycemic patterns of corticosterone release is evidently sex-dimorphic, i.e. absent in female versus stimulatory in male. During hypoglycemia, SF-1 curbs hypoglycemic augmentation of glucagon and corticosterone secretion in the male, but does not exert this action in the female. SF-1 regulation of glucagon release is evidently glucose-dependent as hypoglycemia causes loss of transcription factor control of this hormone profile. Interestingly, hypoglycemia elicits opposite adjustments in GH secretion, namely up- (male) versus down- (female) regulation. Current and previous data support the view that SF-1 may mediate these divergent, sex-specific hormone responses. Ongoing research seeks to characterize the Ghrh neuron neurochemical signals that mediate SF-1 control of GH and glucagon profiles. Data described here show that circulating glucose levels in V- and INS-injected female rats were refractory to VMN SF-1 gene knockdown. As glucose measurements were performed at a one post-INS injection time point, it is possible that additional sampling times, either prior to or after +1 hr, might have disclosed changes in glycemic profiles due to SF-1 siRNA treatment. It is reasonable to presume that circulating glucose concentrations may undergo dynamic change due to VMN SF-1 governance of counter-regulatory hormone release, or control of neural mechanisms governing hepatic gluconeogenic or glycogenolytic functions. Present findings may thus provide a snapshot of a temporal phase during which circulating glucose concentrations are normalized after INS administration as an adaptive reaction to SF-1 - dependent actions that control contra-regulatory outflow.

In summary, current research extends prior research on VMNdm Ghrh neuron SF-1 gene expression with novel evidence that this transcription factor imposes sex-specific control of glucose-regulatory transmitter marker, AMPK catalytic subunit, and ER variant gene expression in this cell population. SF-1 regulation of Ghrh nerve cell expression of genes that encode multiple glucose-regulatory neurochemicals of distinctive chemical structure and spatial/temporal release is sex-dimorphic. SF-1 control of one or more of these co-expressed neurochemicals may contribute to sex differences in neural regulation of glucostasis. There remains a need to establish the role of SF-1—dependent cellular estradiol receptivity and energy sensory function in Ghrh neuron neurotransmission patterns in each sex. Outcomes here identify differential SF-1 regulatory effects on basal and hypoglycemic patterns counterregulatory hormone secretion in the two sexes, including divergent GH outflow in male versus female. Results highlight the need to identify downstream recipients of SF-1—dependent neurochemical transmission and to characterize the functional sequelae of this signaling on neural regulation of glucostasis in each sex.

## Data Availability

The data that support the findings of this study are available from the corresponding author upon reasonable request.

## Declaration of Animal Use

All animal experimental was carried out in compliance with the National Institutes of Health Guide for the Care and Use of Laboratory Animals, 8^th^ Edition, as stated in the submitted manuscript. Sex of animals used is included, along with discussion of sex impacts on study outcomes.

## Statement by Authors

All authors have read the manuscript and have authorized submission for publication, and agree that no changes will be permitted thereafter.

## Submission Declaration

The authors verify that this work has not been published previously, is not under consideration for publication elsewhere, that it is approved by all authors, and that if accepted for publication, it will not be published elsewhere in the same form without written consent of the copyright-holder.

## Nomenclature

ERα: estrogen receptor-alpha
ERβ: estrogen receptor-beta
GAD1/GAD_67_: glutamine decarboxylase (GAD)_67_
GAD2/GAD_65_: GAD_65_
GH: growth hormone
Ghrh: growth hormone-releasing hormone
Ghrh-R: Ghrh receptor
GLS: glutaminase
GPER: G protein-coupled estrogen receptor-1
IIH: insulin-induced hypoglycemia
INS: insulin
nNOS: neuronal nitric oxide synthase
NO: nitric oxide
OVX: ovariectomy
PRKAA1: 5’-AMP-activated protein kinase-alpha_1_
PRKAA2: 5’-AMP-activated protein kinase-alpha_2_
*sc*: subcutaneous
SF-1: steroidogenic factor-1/Nr5a1
VMN: ventromedial hypothalamic nucleus
VMNdm: VMN dorsomedial subdivision
